# Voter Preferences Reflect a Competition Between Policy and Identity

**DOI:** 10.3389/fpsyg.2020.566020

**Published:** 2020-10-15

**Authors:** Libby Jenke, Scott A. Huettel

**Affiliations:** ^1^Department of Political Science, University of Houston, Houston, TX, United States; ^2^Department of Psychology & Neuroscience, Duke University, Durham, NC, United States

**Keywords:** decision making, political science, social cognition, voting, social identity

## Abstract

Canonical rational choice models of voter preferences assume that voters select candidates whose policy positions most closely match their own. Yet, much of the electorate often appears to prioritize identity variables (e.g., social categories, group membership) over policy considerations. Here, we report an empirical test of policy-identity interactions using surveys of likely voters conducted in the 24 hours before the 2016 United States presidential election and the 2018 United States senatorial elections. Each respondent indicated not only their policy preferences but also key social group identities and how those identities would be reinforced by voting. We observed striking evidence for a competition between policy and social group identification: For voters who exhibited the maximal effects of identity, policy positions were essentially irrelevant to their candidate preferences. These results account for dissociations between voters’ stated policy preferences and their voting behavior, while linking empirical observations of political behavior to new models derived from psychology and neuroscience.

## Introduction

Although recently there has been a surge of popular concern about political polarization, political scientists have shown that the American public’s policy positions have not grown increasingly extremist over the past 50 years (Fiorina et al., 2010). The percentage of Americans who classify their ideological affiliation as “moderate/don’t know” remains above 40%, a number that has been stable since the 1970s (Fiorina, 2017). Across a host of policies, from the social safety net to the openness of immigration, the majority of Americans’ preferences remain centrist. This moderation extends even to policies with a moral dimension; for example, the prevalence of a moderate position on abortion has remained stable for the past four decades. Most strikingly, the ability of the left-right dimension to explain policy preferences has actually declined in recent years, suggesting that voters’ policy positions are not polarizing along party lines (Carmines et al., 2012).

Nevertheless, voters increasingly mistrust candidates from the opposing party (Lelkes et al., 2017), express negative stereotypes of those with a different political affiliation (Iyengar et al., 2012), and exhibit partisan animus in job evaluations (Gift and Gift, 2015) and dating behavior (Huber and Malhotra, 2017). This affective polarization may arise from social sorting, such that social group identities increasingly align with party affiliations (Mason, 2016). Following previous political science work (Mason, 2016), by “social group identities” we mean identities that are not based in partisanship,^1^ reflective of other personal identifications. While distinct, social group identities and partisan identities are becoming increasingly correlated. As examples, African Americans and young adults have become more likely to affiliate with the Democratic Party while rural Americans and evangelic Protestants are more likely to affiliate with the Republican Party (Pew Research Center, 2018b). Accordingly, the political divisions observed in modern American society do not necessarily result from divergence in policy positions; instead, associations between social group identities and party affiliations could drive affective polarization – disrupting civil discourse (Rogowski and Sutherland, 2016) and undermining trust in institutions (Iyengar and Krupenkin, 2018). Yet, despite the interest in affective polarization (Rogowski and Sutherland, 2016; Iyengar and Krupenkin, 2018), little attention has been directed toward its potential impact on voters’ candidate preferences themselves.

Building on prior work in neuroscience (Murayama et al., 2010) and psychology (Eisenberger et al., 1999), Jenke and Huettel (2016) theorized a competitive relationship between identity and policy in voter choice. The interaction between economic rewards and socially-based utility has been shown to be competitive in many contexts. In “reward undermining” or “motivational crowding out,” people will devalue socially valued actions when given economic incentives (Frey and Oberholzer-Gee, 1997). The competitive relationship between intrinsic and extrinsic motivating factors has been found using a variety of methods – from behavioral results (Deci et al., 1999) to fMRI studies examining brain signals while people are playing simple games (Frey and Oberholzer-Gee, 1997). Additionally, this competition has been found among a wide variety of behaviors, from sports (Vallerand and Losier, 1999) to economic games (Benabou and Tirole, 2003).

Jenke and Huettel’s model (see Eq. 1) indicates that the utility (*U*_*i,j*_)that Voter*_*i*_* gains by voting for Candidate*_*j*_* results both from the differences between that voter’s and that candidate’s policy positions (*P*_*i*,*x*_−*P*_*j*,*x*_) and from the reinforcement of the voter’s key identities by the act of voting $\left({\hat{I}}_{i,y}-I_{i,y}\right)$. Importantly, they postulated that policy and identity contributions to voter preferences trade off according to a single parameter (δ), such that an increased emphasis on identity would diminish the effects of policy. Though advanced as a theoretical model, it makes quantitative predictions about interactions between voters’ policy positions and identities that can be tested using empirical evidence – and that could suggest interventions based on the rich psychological literature on contributions of identity to behavior. Here, we provide that evidence.

(1) $$U_{i,j}=\delta \,\left(\sum_{x=1}^{n}W_{i,x}\,\left(1-\left|P_{i,x}-P_{j,x}\right|\right)\right)+\left(1-\delta \right)\,\left(\sum_{y=1}^{n}W_{i,y}\,\left({\hat{I}}_{i,y}-I_{i,y}\right)\right)$$

We report the results of empirical assessments of voters’ policy preferences and social group identification, as obtained immediately before the 2016 United States presidential election and 2018 United States midterm election. We collected measures of partisanship, voter engagement, and preferences regarding the common important issues of policy. We also collected information about respondents’ social identification – and about how those identities would be reinforced or undermined by voting for each major-party candidate. We find that our measures of identification robustly predict voters’ preferences, even after accounting for policy positions. And, most critically, we observe strong evidence for the theorized competitive relationship between policy and identity: when identification becomes more important to a voter, policy becomes less important. Our findings are robust: they are computed using a bootstrapping method, have strong effects with small confidence intervals around the reported coefficients, and replicate in two elections. We conclude that in appealing to voters’ identities, candidates may reduce the effect of policy positions on those voters’ choices – a finding with implications for our understanding of political polarization and its potential amelioration.

### Survey Design

The surveys began by asking respondents which candidate they planned to vote for in the upcoming election (see Supplementary Materials for question wording). Participants then indicated their utility for the candidates on “feeling thermometer” scales, which measure how warm or cold a respondent feels toward a candidate. The dependent variable was formed from these questions: it is the respondent’s thermometer score for their favored candidate, ranging from zero to 100 for participants who planned to vote for one of the major party candidates. The order of the next two sections of the survey were randomized to account for potential question order effects, with half of respondents receiving social group identification questions first and the other half receiving policy preference questions first.

We structured our measures of identity based on prior work in social psychology, which sees at least three key dimensions to identification. While measurement techniques have been developed in political science (Huddy, 2001), we took a different approach for two reasons. First, our survey included a large number of social groups and in order to reasonably limit the number of questions it was necessary to skip the measurement of gradations of identification. Secondly, because we are looking distinctly at voting as our dependent variable (rather than the more typically examined dependent variables of political participation, emotional reactivity, or anger), it was important to connect the act of voting, specifically, to the social group identity.

In social psychology, a first dimension of identity is perceiving oneself as similar to prototypical members of the group (Turner et al., 1994). To capture this dimension we used a group-identification question created by Miller et al. (1981). The core of the list of possible groups with which one identifies was adapted from Klar (2013) and Mason and Wronski (2018). Participants answered two questions concerning their social group identification. First, they were given a set of groups and asked to rate whether they felt very close, somewhat close, or not at all close to each of them. For the 2016 election, groups included Whites, the elderly, Blacks, parents, young people, Hispanic Americans, women, the working-class, the middle-class, men, people of your religion, and the military. The 2018 election included the same set, with the addition of Asian-Americans.

Differences may exist between subjective self-categorization and how others would classify an individual. For example, someone with many family members in the military might feel very close to the military though she herself has not served. Thus, we use the term identification instead of identity to capture the subjective nature of our measure. Identification is most relevant to our theory because it is self-generated feelings of closeness that drive individual’s political behavior.

A second key dimension of identity is in-group homogeneity or the extent to which individuals view an entire group as sharing commonalities (Lickel et al., 2000). Our measurement is the percentage of the group perceived by the participant to support the same candidate as she does. Of the groups that respondents ranked “very close,” they then indicated what percentage of that group supports each candidate. We took the average (across all “very close” groups) of the percentage of the group that supported the participant’s preferred candidate. This formed the variable *social distance.* The variable denotes the overall percentage of each respondent’s social groups who support the same candidate; that is, the result provides a measure of the “social distance” of the candidate from the voter’s identified groups.

Last, a third dimension is a sense of coordination with and commitment to fellow members of the group, which should result in participation with in-group activities (Ellemers et al., 1999). This dimension was adapted to the voting context by asking whether participants would feel more or less like a member of their group if they were to vote for their preferred candidate. The variable *social reaction* was formed by taking the average of participants’ answers to this question concerning all very close groups. The variable ranged from zero, “a lot less” close to the group, to four, “a lot more.” Voting is a group action only if the group is relevant to the individual politically, and thus this third dimension excludes those who might be sympathetic toward a group but are not explicitly identified with it. It also establishes the political relevance of the social group identification.

To measure policy preferences, participants were given 14 policy issues (the economy, taxation, foreign policy, health care, gun policy, immigration, social security, education, Supreme Court appointments, treatment of ethnic/racial minorities, trade policy, the environment, abortion, and the treatment of gay, lesbian, and transgender people) and asked to rank them into the three categories of importance. These issues were suggested by Pew Research Polls to be very important to Americans (Pew Research Center, 2016). Of those issues that were reported as “very important,” participants then rated their own preferences and the candidates’ preferences on issue scales. These scales ranged from 0 to 6, with one end point labeled as a very liberal policy and the other labeled with a very conservative policy; there was no labeling of interior points on the scales, following the practices of most political science surveys (e.g., the ANES). This measurement yields how far away the respondent considered the candidate to be from their ideal point, for each of the issues that were important to their vote. The *policy distance* variable was calculated by taking the average of the favored candidate’s distance from the individual’s ideal points across all issues that were “very important” to their vote. This variable was coded such that as the candidate’s proximity to the individual’s ideal point increases, the variable increases. There was a small coding error in the 2016 survey regarding this variable: for two of the identity groups, not everyone who reported being “very close” to them was asked to rate the candidates’ policies. All analyses were repeated both with and without these individuals, and all results replicated (see Supplementary Experimental Procedures). Lastly, participants answered a variety of demographic questions, including the standard seven-level party identification question.

## Results

**FIGURE 1:**
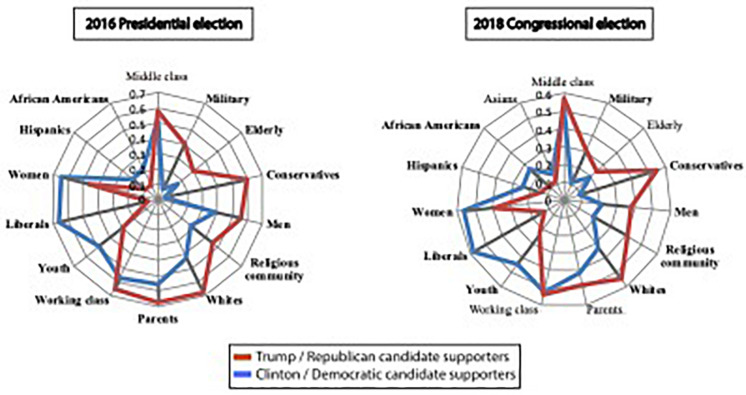
Candidates ‘supporters’ closeness to each social group identity. The proportion of each group of supporters who felt “very close” to each of the social group identities. Boldfaced font indicates identities whose means are significantly different (*p* < 0.05) for supporters of the two candidates. The political identities “Liberals” and “Conservatives” are included as reference points for political partisanship.

We first examined participants’ reported closeness to common social group identities (see section “Materials and Methods”). Prospective voters in 2016 who preferred Donald Trump over Hillary Clinton reported greater affinity (than shown by Clinton voters) for a set of social group identities that included the military, the elderly, their religious community, whites, men, and parents (Figure 1A). Voters who preferred Clinton, in contrast, reported greater affinity for identities such as African Americans, Hispanics, women, and youth. The only social group identity that did not significantly differ between Trump and Clinton supporters was economic: the working class. These results mirror those from a nationally representative sample of verified voters (Pew Research Center, 2018a). Results from the 2018 election (Figure 1B) replicated this pattern. Again, identities like religious communities, whites, and the military were seen as closer by Republican supporters, while identities like women, youth, and African Americans were seen as closer by Democratic supporters – with economic identities among several that did not differ between the parties.

**FIGURE 2:**
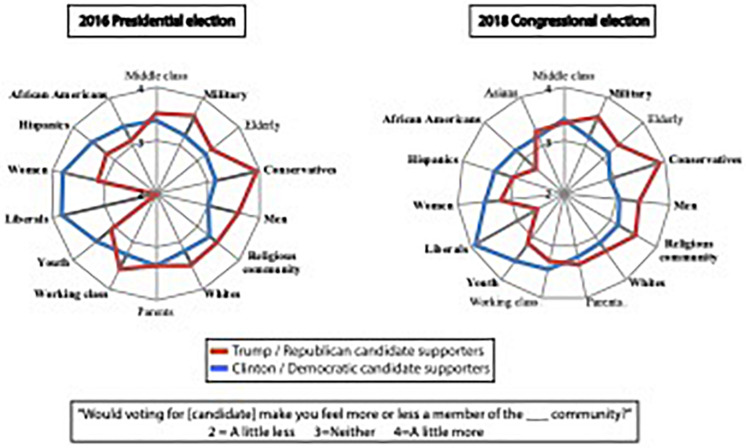
Candidates’ supporters’ average social reaction. Boldfaced font indicates groups whose means were significantly different (*p* < 0.05) for supporters of the two candidates. Note that in the data for the 2016 presidential election, Trump supporters’ mean closeness to the group “liberals” was 1.45 but is displayed as 2 so that the visible range of values in the figure is the same as that of the 2018 Congressional elections, for ease of comparison.

We next evaluated whether participants’ social group identifications were influenced by their upcoming vote. While measures of the degree to which identity is strengthened by voting are not typically collected in political surveys, they could provide important insights into whether identifications are connected to the voting process itself – rather than serving as demographic proxies for political party/issue preferences. Data from both elections provide clear evidence that voting for one’s preferred candidate strengthens one’s most important identifications in a candidate-specific manner (Figure 2); for example, voting for Clinton greatly reinforced *female* or *youth* identities, whereas voting for Trump strengthened *white* or *military* identities. The consistency of this pattern with that observed in Figure 1 supports the inference that identity variables are connected to the act of voting. Yet, stronger evidence will be needed to show that those identifications make contributions that are independent of policy.

For a rigorous test of the role of social group identifications in voter choice, we developed a series of models that progressed from traditional policy distance to include identification and its interactions (Table 1). The factors of these models mapped directly onto terms in Jenke & Huettel (2016). All models attempted to predict the *thermometer ratings* (i.e., liking ratings on a 0–100 scale) of voters’ preferred candidates, as typically used in behavioral political science research (Rahn et al., 1994). We included *partisan extremity* in all models as a control for partisan influences on the strength with which individuals express support; that is, more partisan individuals may express stronger support for their preferred candidate, regardless of other policy or identity variables (Franklin and Jackson, 1983), potentially because party serves as a group identity (Iyengar et al., 2012). However, other research suggests that social sorting, or the linkage between partisan identities and other social group identities (e.g., religious or racial groups), underlies much of this type of polarization (Mason, 2016) – and thus it is critical to consider partisanship alongside other identities in the same model. In our reporting of statistics from these models, we use (2016) to indicate results from the presidential election data and (2018) to indicate results from the midterm election data.

**TABLE 1:** Effects of policy and identity on candidate preferences.

	***DV: Candidate thermometer rating***				
**Formal model variables**	**Empirical model variables**	**Model 1**	**Model 2**	**Model 3**	**Model 4**
	Constant	70.24***	70.24***	70.24***	70.58***
		[68.82, 71.66]	[68.85, 71.63]	[68.76, 71.72]	[69.18, 71.99]
		66.57*** [64.89, 68.25]	66.55***	66.54***	66.86**
			[64.95, 68.15]	[64.90, 68.18]	[65.15, 68.57]
	Partisan extremity	7.51***	6.60***	6.41***	6.39***
		[6.02, 9.00]	[5.06, 8.14]	[4.85, 7.98]	[4.83, 7.94]
		12.02***	11.20***	11.13***	11.35***
		[10.12, 13.86]]	[9.33, 13.08]	[9.25, 13.00]	[9.43, 13.26]
(|*P*_*i*,*x*_-*P*_*j*,*x*_|)	Policy distance	7.12***	6.73***	6.27***	5.92***
		[5.29, 8.95]	[4.97, 8.49]	[4.57, 7.98]	[4.27, 7.58]
		6.14***	5.66***	5.49***	5.18***
		[4.15, 8.14]	[3.70, 7.62]	[3.61, 7.38]	[3.34, 7.01]
	Social distance		3.88***	2.72**	2.74**
			[2.34, 5.42]	[1.16, 4.28]	[1.27, 4.21]
			4.15***	3.29***	3.27***
			[2.37, 5.93]	[1.49, 5.09]	[1.48, 5.06]
(${\hat{I}}_{i,y}-I_{i,y})$	Social reaction			3.79***	4.17***
				[2.33, 5.25]	[2.59, 5.74]
				2.15*	2.44**
				[0.35, 3.95]	[0.73, 4.15]
(δ)	Policy × social reaction				−1.88*
					[−3.32, −0.45]
					−2.17*
					[−3.93, −0.40]
	Adjusted *R*^2^	0.23	0.26	0.28	0.28
		0.32	0.34	0.35	0.35
	*N*	852			
		608			

The effects of policy and identity variables on candidate preferences (i.e., thermometer ratings) in the 2016 presidential election (rows with a white background) and 2018 midterm elections (rows with a gray background). *p < 0.05, **p < 0.01, ***p < 0.001. 95% confidence intervals are shown under the coefficients. Formal model variables were drawn from Equation 1, as proposed by Jenke and Huettel (2016).

We first replicated traditional rational choice models by examining the effect of *policy distance* (Model 1). Every participant rated each of 12 issues according to the importance for their vote; for each issue rated as “very important,” the participant placed their own position and their preferred candidate’s position on a policy scale. The *policy distance* measure was defined as the absolute value of the difference between those two positions for those key issues. As expected (Iyengar and Krupenkin, 2018), we found that *partisan extremity* was a strong predictor of thermometer ratings, such that as a voter’s partisanship increases, their evaluation of their preferred party’s candidate also increases. Moreover, we found that candidates in both elections were rated more favorably when their positions were close to the participant’s ideal positions [i.e., minimum *policy distance*; (2016) β = 7.12, *p* < 0.001 and (2018) β = 6.14, *p* < 0.001]. These results fit the predictions of spatial models of voter choice (Jenke and Huettel, 2016).

Next, we incorporated social identifications into our model (Model 2). We asked participants to indicate their affiliation with different identities (cf. Figure 1); for those identities judged to be very close to their own, the participant estimated the percentage of that group who supported their own favored candidate. For example, if a Clinton supporter indicated a close affiliation with *female* and *working class* identities, she would be asked to estimate the percentages of females and of working class individuals who supported Clinton. Our *social distance* measure was defined as the average percentage across all such very close identifications.

We found that *social distance* was a significant predictor of voter preferences in both elections [(2016) β = 3.88, *p* < 0.001 and (2018) β = 4.15, *p* < 0.001] – and the increase in r-squared indicates that its inclusion explained additional variance beyond that of the previous model, while both *policy distance* and *partisan extremity* remained significant. This finding shows that voters tend to prefer candidates who are supported by other voters similar to themselves. This result is in line with previous results found by political psychologists regarding the effect of political party (Green et al., 2004), ethnicity (Jackson, 2011), and gender (Sanbonmatsu, 2004) on voter choice.

Notably, the results from Model 2 also extend previous findings that closeness to social groups that are aligned with political parties and increases in partisan identity together lead citizens to express increased affective attachment to their party. The strength of both variables increases not only affective attachment to copartisans but also more favorable ratings of the party’s presidential candidate. However, it does not show that the contribution of social distance is independent from policy distance, since members of a group may vote for the same candidate due to shared policy concerns.

The model in Eq. 1 makes the additional prediction, however, that the act of voting for a favored candidate actually strengthens or weakens social group identifications – and thus changes in those social group identifications contribute independently to the utility derived from voting. To test this prediction, we took the same “very close” identities used to construct the *social distance* measure and then asked participants how voting for their preferred candidate would strengthen or weaken their affiliation with those identities. Our *social reaction* measure was defined as the average response across those very close identities. For example, someone who reinforces their *conservative* and *military* identifications by voting for Trump would have a high *social reaction* value. We found (Model 3) that *social reaction* was a significant positive predictor of voter preferences in both elections [(2016) *p* < 0.001 and (2018) *p* < 0.05] such that its inclusion improved the overall predictive power of the model, while all terms from the previous model remained significant. This result is consistent with the prediction that voter preference for a candidate depends on how the act of voting for that candidate strengthens social group identifications.

Note that high levels of congruence theoretically could occur between identity affiliation and policy stances, making it difficult to identify the causal mechanism at the root of the relationship with voter choice. For example, an individual might have a liberal stance on equal pay laws because she strongly identifies with the social category of “women”; alternatively, her identification with women when voting might reflect her underlying policy positions. We used a variance inflation factor analysis to demonstrate that the variables used in Model 3 were not collinear in our data, providing confidence in our interpretation of their coefficients (Supplementary Table S2).

**FIGURE 3:**
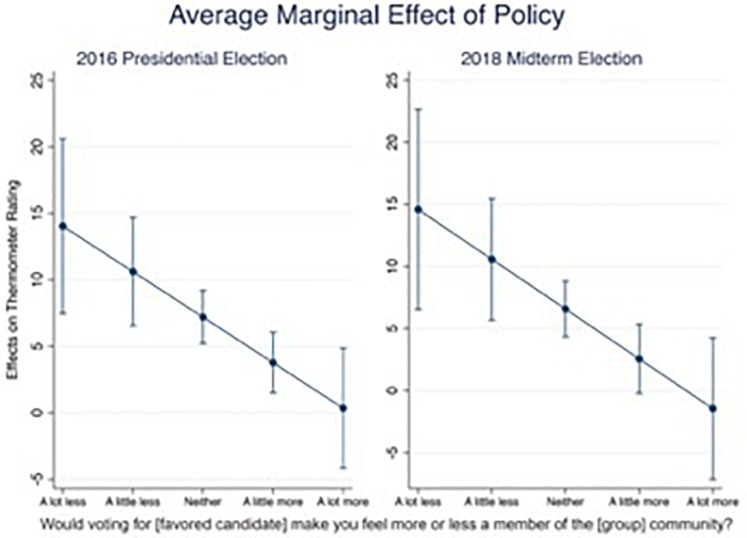
The average marginal effect of policy on candidate ratings. The average marginal effect of policy on candidate ratings, across levels of social reaction. Vertical lines show 95% confidence intervals.

Finally, we tested the prediction (Model 4) that there should be a significant interaction (represented by δ in Eq. 1), such that as the effect of identification increases (i.e., larger effect of *social reaction*) there should be a diminishing effect of policy on voter preferences (i.e., smaller effect of *policy distance*). This prediction was borne out in our data both for the 2016 election [*p* < 0.05] and for the 2018 election [*p* < 0.05]. To assess the effect size of this interaction variable alongside the effects of *policy distance* and *social reaction*, we examined the average marginal effect of *policy distance* across values of *social reaction*, holding all other variables at their means (Figure 3). Among voters in each election who reported that their identification was not influenced by voting, policy distance had an approximately 15-unit effect on their candidate ratings; for comparison, that effect is roughly twice the magnitude of *policy distance* in Model 1. However, among those who indicated that voting for their preferred candidate would have the strongest positive effect on their identification, the average marginal effect of policy was not significantly different from zero. In other words, for those individuals whose votes were most driven by identification, policy positions played no role in their ratings of candidates. These effects were consistent across the two elections. We interpret these results as very strong evidence that identification and policy compete in determining voter preferences^2^.

Across the four reported models, almost all coefficients from the 2018 election are within the 95% confidence interval of those from the 2016 election and vice versa, indicating the good replicability of these effects. Yet the confidence intervals for the coefficients on *partisan extremity* do not overlap between the two elections on any of the models. Those from the 2018 midterm election are consistently higher than those from the 2016 presidential election – a finding consistent with prior demonstrations that in Congressional elections voters know less about candidate’s ideological preferences and thus rely more on party identification, relative to presidential elections (Mann and Wolfinger, 1980).

## Discussion

How identity influences voters’ preferences has received increasing attention both in political psychology and in the popular media. While identity’s effect on political behavior was first considered within political science almost six decades ago (Campbell, 1960), the identities traditionally considered were almost exclusively limited to party identification, with other racial, ethnic, and religious identities being less frequently investigated. More recent research can be separated into two main strands.

One strand uses experimental approaches to explore how social groups arise and how group members perceive themselves and others, using groups beyond political party like parental status (Klar, 2013), sexual orientation (Egan, 2012), and race (Sigelman et al., 1995). Such studies examining identity often rely on demographic information to measure identity – usually limited to a single social category – which poses challenges for distinguishing identities from related policy positions (Rabushka and Shepsle, 1972). As a result, such studies rarely examine voter choice as the dependent variable, choosing instead to examine voter participation or measures of affective polarization including anger, enthusiasm, and party support. Our multiple measurements of voter social group identification – including both as a proxy for party and interactions with the act of voting itself – account for potential bidirectional causation between identity and policy variables.

A second line of research models the effects of identity using formal theory to specify voters’ utility functions that include non-economic, identity concerns – which in turn could be primed strategically by politicians (Akerlof and Kranton, 2000; Dickson and Scheve, 2006). While useful in elucidating the process by which identity could affect voting behavior, such models are rarely empirically tested. Moreover, such tests could prove difficult, since they typically leave the definition of identity abstract, rather than assessing identities through concrete empirical means.

This paper addresses the gap between these two strands of research by taking a generalizable model of the effects of identity (Jenke and Huettel, 2016) and demonstrating that this new model improves predictions over the traditional determinants of candidate choice (i.e., policy distance). Our survey questions allowed us to pinpoint the effect of individuals’ social group identification on voting, thus distinguishing whether voters’ expressed identities served as proxies for policy positions or as independent and potentially competitive contributors to their preferences. This approach provides a foundation for further integration of empirical and formal work on the effects of identity upon voter preferences.

Our results have surprising implications for models of voter choice: Identity variables cannot simply be added to models alongside policy measures, but instead interact with those policy measures in a competitive manner. These results provide the first empirical test of the model of identity and policy advanced by Jenke and Huettel (2016), who contend that when identity exerts a major influence, policy makes minimal contributions to determining a voter’s choice. By showing that policy essentially has no impact on candidate evaluation when social group identification plays a strong role (Figure 3), the current data corroborate the predictions of that model. The observed competition between policy and identification is consistent with previous findings in psychology and neuroscience showing that economic and social rewards do not additively combine in many contexts (Eisenberger et al., 1999; Murayama et al., 2010). By extending these concepts to political behavior, our results suggest the viability of consilient explanations for social science phenomena that span levels of analysis from neuroscience through lab behavior to real-world empirical findings.

Questions remain regarding the intersection of policy and identity. Consider the proposed neural mechanism behind our hypothesis: social relationships like identity are tracked when a specialized brain system becomes engaged (Carter and Huettel, 2013; Scheepers and Derks, 2016). If correct, this mechanism suggests several corollary effects of identity on voting. Most notably, as a single identity becomes more salient, *all* identities should increase in their impact on candidate evaluation while all policies becomes less important. This suggests that priming only one aspect of one’s political identity is sufficient to de-emphasize policy – a suggestion not yet tested. Methods of psychology and neuroscience that provide information about underlying mechanisms (Jost et al., 2014) – such as functional MRI, electroencephalography, or eye-tracking – may provide important information about the process of competition while avoiding the limitations of traditional approaches (e.g., self-report bias).

Our results also offer an explanation for recent political events. Before the Brexit vote, economists and British voters alike agreed that a “leave” vote would have catastrophic economic impacts. The implicit reframing of the Brexit vote as a referendum on national identity, however, may have primed voters’ own identity-related concerns, diminishing the impact of any concerns about the economy or other related policy issues (Matti and Zhou, 2017). Similarly, identity may have shaped voters’ preferences in the 2016 United States presidential election. For example, white respondents who were high in ethnic identification responded with increased support for Trump when reminded that projections predict white Americans will become a minority by 2042 (Major et al., 2018). Our study suggests that other identities – e.g., the military, religion, and the elderly – may have been reinforced by support for Trump, drawing voters who might have otherwise disagreed with his policy positions. However, our findings were not specific to 2016 but also generalized to the subsequent 2018 Congressional election – indicating that identity remained an important contributor to voter choice thereafter.

We note that identity is flexible and subject to priming effects (Huddy, 2001), which could make appeals to identity an effective tool of political campaigns. Contextual cues may push individuals toward voting based on only the affective polarization associated with increased social group identity or solely on the relative centrism associated with issue positions. Appeals to voters’ identities could be especially beneficial for candidates whose policy positions are distant from those of many voters (e.g., incumbents who have long-established positions on now-controversial issues) but could backfire for candidates whose policy positions are more representative of the electorate. Conversely, candidates whose social group identity is mismatched to many voters – or whose opponent’s identity does resonate with voters – would benefit from emphasizing policies instead. Strategies that emphasize identity may be particularly powerful because they first shape how voters think about themselves – and subsequently alter how those voters evaluate *all* candidates in an election.

Last, our study has implications for understanding the paradox of policy moderation but affective polarization. When voters’ identities are the key driver of their candidate choices, affective polarization may contribute to implicit partisan prejudice and also become a key arbitrator of vote choices. However, when policy positions are foremost in their minds, voters are unlikely to apply their more polarized affect to the decision and may rely instead on their more moderate policy positions. Our results suggest that the turmoil and animosity predicted by affective polarization may have deleterious effects on civil discourse, but do not necessarily undermine the act of voting itself.

## Materials and Methods

### Samples and Data Collection

We collected our sample for the 2016 United States presidential election via Amazon’s Mechanical Turk (MTurk) from 3:41 p.m. on November 7, to 9:41 a.m. on November 8, 2016, the date of the United States presidential election. Participants were paid $0.50 for their participation, and the average completion time for the survey was 7 min and 53 s. The majority of responses were from November 7 (only 84 responses were from November 8). Those who answered attention check questions incorrectly (*N* = 171), preferred to abstain or vote for a candidate other than Trump or Clinton (*N* = 226), or listed no issues as “very important” or no groups as “very close” (*N* = 35) were dropped. Our final sample contained the remaining 852 respondents, all over age 17. The approving IRB board was Duke Campus IRB.

For the 2018 United States midterm election, our sample was collected via MTurk from 10:15 a.m. on November 5th until 5:00 a.m. on November 6th, 2018. Participants were paid $0.75 and the average length of the survey was 9 min and 9 s. Respondents who preferred to vote for a candidate other than the Democratic or Republican senate candidate (*N* = 161) or listed no issues as “very important” or no groups as “very close” (*N* = 8) were dropped from the sample. Additionally, those who indicated that they used outside sources (such as the internet) to help them answer questions or answered randomly rather than thinking about their answer were excluded (*N* = 64). These exclusions left us with a final sample size of 608. Half of the data was obtained from states that were predicted to have close races by FiveThirtyEight.com: Arizona, Mississippi, Nevada, Missouri, Florida, Montana, and Indiana. The other half was obtained from states that were predicted solidly or likely in favor of one of the parties. S1 contains details of the characteristics of the two surveys’ samples. The data and code reproducing our tables are available at http://ljsurvey1.us/sdm_downloads/identitypolicy-2/ and can be downloaded under the password “pnasIdentityPolicy.” The data will be made publicly available without password protection upon publication.

All procedures were conducted under a protocol approved by the Institutional Review Board of Duke University. Our experiment was opened for enrollment to all participants on the MTurk platform for the defined time periods. Since our theoretical model was published before empirical data collection, there was not prior data regarding the anticipated effect size. Thus, we adopted a procedure of open-ended enrollment with a strong replication requirement across the two data sets.

### Statistical Analysis

Models 1, 2, 3, and 4 were run using a non-parametric bootstrapped ordinary least squares regression. Bootstrapping builds a sampling distribution by resampling the data (in this case, 1,000 times), drawing randomly from it with replacement. The results of the model were then averaged over all these samples. We standardized all independent variables in the model, such that they had a mean of zero and a standard deviation of one.

## Data Availability Statement

The datasets presented in this study can be found in online repositories. The names of the repository/repositories and accession number(s) can be found below: http://ljsurvey1.us/sdm_downloads/identitypolicy-2/ Password: pnasIdentityPolicy.

## Ethics Statement

The studies involving human participants were reviewed and approved by Duke University IRB. Written informed consent for participation was not required for this study in accordance with the national legislation and the institutional requirements. Written informed consent was implied via completion of the survey.

## Author Contributions

Both authors developed the study concept and design, drafted the manuscript, and approved the final version of the manuscript for submission. Data collection and analysis were performed by LJ, with input from SH.

## Conflict of Interest

The authors declare that the research was conducted in the absence of any commercial or financial relationships that could be construed as a potential conflict of interest.

## Footnotes

1 However, note that political parties can be considered an exemplar of the broader term “social identity” in that they are a group with which citizens may have a sense of shared background, values, and/or identity.

2 See Supplementary Figure S3 for the marginal effect of *social reaction* across levels of closeness on policy.
