# Supplementary material for: Voter Preferences Reflect a Competition Between Policy and Identity
Source: Front Psychol. 2020 Oct 15;11:566020. doi: 10.3389/fpsyg.2020.566020 (PMC7593652; doi:10.3389/fpsyg.2020.566020)
Supplement: Supplementary file 1 [file Data_Sheet_1.docx]

**Supplementary Materials**

S1: Characteristics of the surveys' samples

| **Variable** | **Mean and standard deviation, 2016 presidential election** | **Mean and standard deviation, 2018 midterm election** | **Question wording** | **Coding** |
| --- | --- | --- | --- | --- |
| **Party identification** | 2.02 [0.96] | 2.02 [0.94] | (1) Generally speaking, in politics do you consider yourself . . .  (2a) Would you call yourself a "strong" or "not very strong" Democrat/Republican?  (2b) Do you think you yourself as closer to the Republican or Democratic Party? | 1=Strong Democrat; 2=Not-so-strong Democrat; 3=Leaning Democrat; 4=Independent; 5=Leaning Republican; 6=Not-so-strong Republican; 7=Strong Republican |
| **Political interest** | 3.31 [0.66] | 2.17 [0.97] | Generally speaking, how interested are you in politics and public affairs? | 1=Not interested at all; 2=Not very interested; 3=Somewhat interested; 4=Very interested |
| **Political knowledge** | 3.93 [1.01] |  | Indicate which is true about you in response to the statement, “I feel like a have a pretty good understanding of politics" | 1=Disagree strongly; 2=Disagree somewhat; 3=Neither agree nor disagree; 4=Agree somewhat; 5=Agree strongly |
| **Political knowledge** |  | 2.23 [0.87] | 1. Who is the current speaker of the U.S. House of Representatives?  2. How long is the term of office for a U.S. Senator?  3. What job or political office is now held by John Roberts?  (Each question has five possible answers, including 'Don't know') | The sum of correct answers across the three questions |
| **Race** | 0.77 [0.42] | 0.77 [0.42] | What racial or ethnic group or groups best describe you? | 0=Non-white; 1=White |
| **Education** | 3.61 [0.86] | 3.59 [0.83] | What is the highest degree you have earned? | 1=Grade school; 2=High school; 3=Some college; 4=College; 5=Advanced degree |
| **Income** | 1.80 [0.62] | 1.78 [0.59] | Was your total household income in the past twelve months . . . | 1=Less than $35,000; 2=Between $35,000 and 100,000; 3=Greater than 100,000 |
| **Age** | 3.21 [1.18] | 3.52 [1.18] | What is your age? | 1=Less than 18; 2=18-29; 3=30-39; 4=40-49; 5=50-60; 6=More than 60 |

S2: Test for collinearity across variables

| Variable | VIF | 1/VIF |
| --- | --- | --- |
| Policy | 1.10 | 0.91 |
|  | 1.08 | 0.93 |
| Social distance | 1.19 | 0.84 |
|  | 1.26 | 0.79 |
| Social reaction | 1.15 | 0.87 |
|  | 1.22 | 0.82 |
| Partisanship | 1.14 | 0.88 |
|  | 1.10 | 0.91 |

Supplementary Table 2: Variance inflation factors for the variables used in Model 3. Estimated coefficients from the 2016 presidential election are shown on rows with a white background, while results from the 2018 midterm elections are shown on rows with a grey background. We observed low variance inflation factors (all < 2), indicating that our model does not suffer from multicollinearity.

S3: The average marginal effect of social reaction at different distances from the candidate

The average marginal effect of social reaction, across levels of policy distance. Vertical lines show 95% confidence intervals.

**Question wording**

Candidate preference:

If you were to vote in the upcoming Senate election in (state name), who would you vote for?

- (Republican candidate’s name)
- (Democratic candidate’s name)
- Someone else
- Don’t know

I’d like you to rate (Republican candidate) and (Democratic candidate) using a “feeling thermometer” scale, shown below. The higher the number, the warmer or more favorable you feel towards him or her. The lower the number, the colder or less favorable you feel.

Policy placements (shown with health care as an example policy):

Please indicate where you would place your own preference, (Republican

candidate’s name)’s preference, and (Democratic candidate’s name)’s preference on health care using the sliding scales below. On the scales, 0 indicates, “a government insurance plan” and 100 indicates, “a private insurance plan”. And the lines in between indicate a preference somewhere between the two.

First dimension of identification

Here is a list of social groups. Please read over the list and then indicate which groups you feel very close to by moving those groups into the box on the right hand side. By “very close,” we mean people who are most like you in their ideas, interests, and feelings about things.


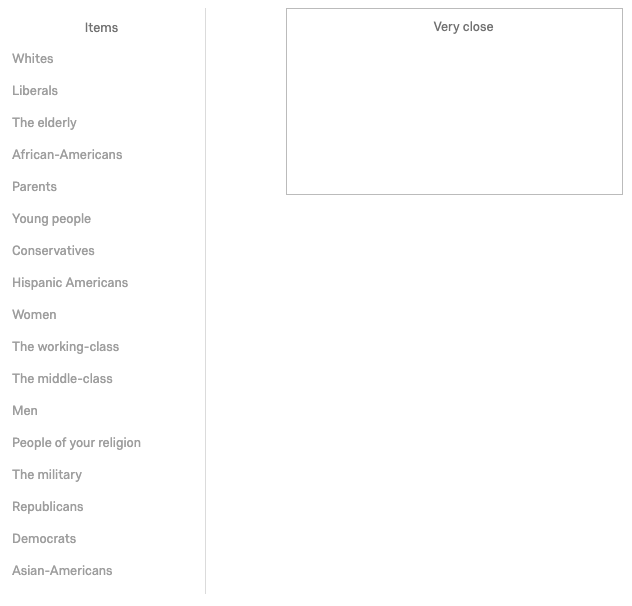


Second dimension of identification (Example: Rhode Island and the military community)

How is support for the two major party candidates distributed amongst the military people who you know? Considering Robert Flanders and Sheldon Whitehouse, what percentage supports each candidate? Please indicate your answer using the slider below.


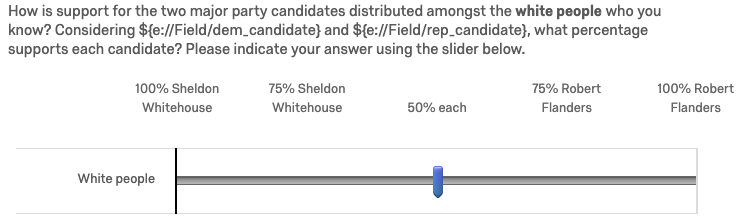


Third dimension of identification (Example: the military community)

Would voting for (preferred candidate) make you feel more or less a member of the military community?

- A lot more
- A little more
- Neither
- A little less
- A lot less

**Data quality: Examining the effects of a data coding error**

Subsequent to data collection, we identified an error in the coding of the survey that affected the issues displayed to some of our participants. Specifically, after each participant rated the importance of each policy issue, the survey then selected those issues rated as “very important” for follow-up questions about policy distance. The coding error influenced the display of two of the issues (“treatment of LGBT individuals”) and (“treatment of ethnic minorities”). For the former issue, the follow-up question about policy distance was not asked of 119 participants (of 311 who indicated “very important”) and for the latter issue the follow-up question was not asked of 172 participants (of 352 possible). We note that for all other participants and for all other issues, the policy distance questions were included appropriately – and these participants were still asked about policy distance for other issues important to them.

We repeated our main model for each of the above subsets of participants: (1) the 311 participants indicating LGBT rights as “very important” and (2) the 352 participants indicating treatment of ethnic minorities as “very important.” For each subset, we evaluated separate models for those participants asked the follow-up questions and those not asked the follow-up questions. We wanted to rule out the possibility that data from those participants who were not asked the full set of follow-up questions were biasing our results. For both subsets and for all terms in our main model, all coefficients were in the same direction in the asked and not-asked groups. Moreover, there were no terms for which an effect of interest was significant in the not-asked group but non-significant in the asked group.

We conclude that this coding error could not bias results for these specific policy issues, much less alter the results from the full set of policy issues. Furthermore, this coding error was not present in the 2018 experiment, which replicated all significant results from the 2016 model.
